# Development and Evaluation of 4 Short, Animated Videos for Women in Midlife Promoting Positive Health Behaviors: Survey Study

**DOI:** 10.2196/60949

**Published:** 2024-12-02

**Authors:** Karin Hammarberg, Mridula Bandyopadhyay, Hau Nguyen, Flavia Cicuttini, Karin Andrea Stanzel, Helen Brown, Martha Hickey, Jane Fisher

**Affiliations:** 1 School of Public Health and Preventive Medicine Monash University Melbourne Australia; 2 Department of Obstetrics Gynecology and Newborn Health University of Melbourne Melbourne Australia; 3 School of Exercise and Nutrition Sciences Deakin University Melbourne Australia

**Keywords:** health promotion, healthy aging, self-management, midlife, menopause, internet, video, animation, survey, questionnaire, education, women, gynecology

## Abstract

**Background:**

Health and health behaviors in midlife are important determinants of healthy aging. There is evidence of unmet needs for health-promoting information for women from culturally and linguistically diverse backgrounds and women with low literacy.

**Objective:**

This study aimed to (1) develop accessible short, animated videos viewable and downloadable from YouTube aimed at promoting positive health behaviors in women in midlife and (2) evaluate their accessibility, acceptability, understanding, and usability and whether this was influenced by the level of education or socioeconomic disadvantage.

**Methods:**

In collaboration with a video production company, a multidisciplinary team of academics and health professionals developed 2 short, animated videos on self-management of menopause health and 2 promoting joint health. Their accessibility, acceptability, understanding, and usability to women were evaluated in an anonymous web-based survey.

**Results:**

A total of 490 women viewed the videos and responded to the survey. Of these, 353 (72%) completed all questions. Almost all (from 321/353, 91% to 334/363, 92%) agreed that the information in the videos was “very easy to understand.” The proportions reporting that all or some of the information in the video was new to them varied between videos from 36% (137/386) to 66% (233/353), the reported likelihood of using the practical tips offered in the videos varied from 70% (271/386) to 89% (331/373), and between 61% (235/386) and 70% (263/373) of respondents stated that they would recommend the videos to others. Education-level group comparisons revealed few differences in opinions about the videos, except that women with lower education were more likely than those with higher education to state that they would recommend the 2 joint health videos to others (36/45, 80% vs 208/318, 65%; *P*=.051 for video 3; and 36/44, 80% vs 197/309, 64%; *P*=.04 for video 4). There were no differences between women living in the least advantaged areas (Socioeconomic Indexes for Areas quintile areas 1 and 2) and those living in the most advantaged areas (Socioeconomic Indexes for Areas quintile areas 3, 4, and 5) in their responses to any of the questions about the 4 videos.

**Conclusions:**

Most women found the videos easy to understand, learned something new from watching them, planned to use the practical tips they offered, and were likely to recommend them to other women. This suggests that short, animated videos about health self-management strategies in midlife to improve the chance of healthy aging are perceived as accessible, acceptable, easy to understand, and useful by women.

## Introduction

Recent years have seen a shift in public discourse relating to menopause, which has moved from the portrayal of menopause as a debilitating and unwelcome life phase characterized by endocrine deficiency to viewing it as a biological transition that women can manage with the right support. Hickey et al [1] advocate for an empowerment model to support women transitioning into menopause and beyond, where empowerment is defined as an active process of gaining knowledge, confidence, and self-determination to self-manage health and make informed decisions about care.

In high-income countries, women’s life expectancy is now around 85 years [2] and women over the age of 50 years are one of the fastest-growing demographic groups [3]. It is well established that health and health behaviors in midlife are important determinants of healthy aging [4]. In women, midlife usually coincides with the menopausal transition, a life stage when physiologic changes occur that are linked to bone, cardiovascular, cognitive, and musculoskeletal health [4]. Furthermore, as populations globally are aging, the proportion of people with osteoarthritis is increasing [5]. The joint pain caused by osteoarthritis can be debilitating, and studies show that anxiety and depression are common among patients with this condition [6]. Postmenopausal weight gain is common, and a recent systematic review concluded that weight gain in adults is associated with an increased risk of knee osteoarthritis [7].

Healthy aging is governed by complex interactions between biological, environmental, socioeconomic, and cultural factors [8-10]. Some of these factors are beyond personal control. Others, including smoking, poor nutrition, weight gain, and lack of physical activity, are potentially modifiable. Because these factors are associated with the development of chronic conditions such as type 2 diabetes, joint pain, and cardiovascular disease, it is important to improve health literacy relating to self-care strategies for optimizing health in midlife to increase the chance of healthy aging [9,11].

Health literacy is the capacity “to obtain, process, and understand health information and services needed to make appropriate health decisions” [12]. Socioeconomic disadvantages, including having a low income, low educational attainment, and low literacy and being a nonnative English speaker, are associated with lower health literacy and poorer health [13,14].

The internet is increasingly used to access health information. While access to the internet has been shown to reduce health inequalities [15], the readability of web-based health information is mostly above the average reading level [16,17], making it inaccessible to people with low literacy and those with English as a second language [18].

We used content analysis to assess the accessibility of the information on 16 Australian websites with health information for women in midlife and found that most offered written factsheets with content above the average adult reading ability, only 4 included written resources in languages other than English, and only 2 had information in audiovisual format in languages other than English. We concluded that more accessible information resources for health self-management to reduce the risk of chronic disease are needed for women in midlife for whom English is not their first language and those with low literacy [19].

Digital health promotion resources have the potential to reach many people from diverse backgrounds with health-promoting messages [20]. Based on the evidence of unmet needs for health-promoting information for women from culturally and linguistically diverse backgrounds and women with low literacy, this study aimed to (1) develop accessible short, animated videos viewable and downloadable from YouTube aimed at promoting positive health behaviors in women in midlife and (2) evaluate their accessibility, acceptability, understanding, and usability and whether this was influenced by level of education or socioeconomic disadvantage.

## Methods

### Video Development

A multidisciplinary team of academics and health professionals with extensive research, professional, and personal experience of menopause and healthy aging developed the template for short, explanatory, animated videos with practical evidence-informed tips about maintaining good health in midlife. In addition to their research experience, team members have clinical expertise in psychology, rheumatology, health promotion, gynecology, and women’s health nursing.

The decision to use narrated animated videos was based on a review of 38 trials demonstrating the efficacy of this medium in improving knowledge about medical procedures, management of chronic conditions, health promotion, and disease prevention [21]. In addition, evidence from a study of colorectal cancer screening found that people with low health literacy recalled as much information as their more health-literate counterparts when given the opportunity to watch spoken videos [22].

After reviewing the evidence about menopause and potentially modifiable factors in midlife that can affect women’s future health trajectory, the team decided to create four 2-minute videos covering (1) explanation of the physiology of menopause, common menopause-related symptoms and how they can be managed, positive aspects of menopause, and a recommendation to seek medical advice if symptoms are severe (“What is menopause?”); (2) practical steps women can take to optimize their health during the menopausal transition and in later life (“How to stay healthy after menopause”); (3) practical advice on managing joint and back pain (“Slowing weight gain to reduce joint pain”); and (4) simple strategies to reduce joint pain by avoiding weight gain (“Maintaining weight for joint health”).

The development of the video content was informed by a review of the literature, which identified “...focus on enjoyment, health benefits including healthy aging, social support, clear messages, and integration of behaviors into lifestyle” as facilitators for the uptake and maintenance of positive health behaviors by people in midlife [23].

After expert advice from an animated explanatory video production agency, Punchy Digital Media [24], that videos should ideally be up to only 2 minutes long and have fewer than 300 spoken words, draft scripts were developed with particular attention to simple language (Australian education year 8 readability) and short sentences, framing messages in a clear and positive way and offering advice about health behaviors that can be integrated into daily life [23]. The scripts were then edited by the agency’s experts, and after several rounds of feedback from researchers, a final script was agreed on. Using these scripts, the agency developed a storyboard for the narration of each video in consultation with the research team. The storyboards underwent multiple iterations in response to the researchers’ comments until a consensus was reached. The animations were then created in an iterative process where feedback from the researchers was integrated until a final version was agreed on. The videos were produced using traditional 2D motion graphics. This style uses flat 2D illustrations, icons, typography, shapes, and container elements brought to life through motion and transitions. Animated characters guide viewers through concepts step-by-step as infographics pop on screen to visually illustrate spoken messages. The final animations had images of women with different body shapes, ages, skin color, and clothing conveying ethnic and cultural diversity.

As the target populations included women from culturally and linguistically diverse backgrounds, the services of Ethnolink [25], an expert multicultural and multilingual communications agency used by government and health organizations across Australia, were sought. They translated the scripts into Vietnamese and Simplified Chinese, the 2 most commonly spoken non-English languages in Australia, and tested the translations and animations with women from these language backgrounds. Some changes in the captions and voice-over were made in response to their feedback.

### Evaluation of the English-Language Version of the Videos

In this study, data were collected through an anonymous web-based survey.

#### Materials

The English versions of the educational videos were embedded in an anonymous web-based survey to ascertain women’s perspectives on the accessibility, acceptability, understanding, and usability of the videos. The participants were asked to view the four videos sequentially and answer four questions after each: (1) How easy was it to understand the information in the video? (very easy, quite easy, quite hard, and very hard); (2) How new was the information in the video to you? (the information was completely new, some of the information was new, a bit of the information was new, and none of the information was new); (3) How likely are you to use some of the tips from the video? (very likely, quite likely, not very likely, and not likely at all); and (4) Would you recommend this video to others? (yes, maybe, and no). After viewing the 4 videos and answering the questions, respondents were asked to state their age, highest level of completed education (no formal education, primary school [years 1-6], secondary school [years 7-12], and postschool qualification), state of residence, postcode, and main language. The survey was administered using Qualtrics software [26].

#### Participants and Recruitment

Women aged 18 years or older were eligible to participate. They were recruited through advertising on social media platforms (Facebook [Meta] and Twitter [rebranded as X]) and Jean Hailes for Women’s Health’s newsletter [27]. Jean Hailes for Women’s Health is the national digital gateway for women’s health funded by the Australian Government. The advertisement included detailed participant information and links to the videos and survey questions.

### Data Management and Analysis

Responses entered by respondents in Qualtrics were downloaded into SPSS version 28 (IBM Corp) [28]. Responses to the questions about the videos were dichotomized: (1) ease of understanding information: very easy vs quite easy, quite hard, and very hard; (2) how much of the information was new: the information was completely new and some of the information was new vs a bit of the information was new and none of the information was new; (3) likelihood of using the tips from the videos: very likely and quite likely vs not very likely and not likely at all; and (4) likelihood of recommending videos to others: yes vs maybe and no. Postcodes were used to determine Socio-Economic Indexes for Areas (SEIFA) quintile. SEIFA is a ranking of areas in Australia according to relative socioeconomic advantage and disadvantage. SEIFA quintile 1 represents the most disadvantaged and quintile 5 the most advantaged areas. Respondents were grouped by level of education (primary and secondary school vs postschool qualification) and socioeconomic advantage (SEIFA 1 and 2 vs SEIFA 3, 4, and 5). The cutoff points for dichotomized variables were chosen to provide as equal-sized groups as possible. Data were analyzed using descriptive statistics and chi-square test for group differences between those with low versus high levels of education and those in the more (SEIFA 1 and 2) and less (SEIFA 3, 4, and 5) socioeconomically disadvantaged groups. Not all respondents answered all questions; the numbers who did are shown for each question in the tables.

### Ethical Considerations

The study was approved by Monash University’s Human Research and Ethics Committee (2023-39025-93342). Participant information about the purpose of the study and the anonymous nature of the survey was provided before potential participants were directed to the survey. The participant information also stated that completion of the survey questions was taken as consent to participate in the study. In recognition of their time, women who completed the survey were offered the opportunity to enter a draw for 1 of 10 Aus $40 (Aus $1=US $0.64) gift cards. Those who wanted to participate in the draw were asked to provide an email address in a file not linked to their survey responses.

## Results

In all 490 women responded. Of these, 353 (72%) completed all questions. Respondent characteristics are shown in Table 1.

**Table 1:** Respondent characteristics.

Characteristics		Value
**Age (years; n=452), mean (SD)**		53.5 (8.8)
**Level of education (n=446), n (%)**		
	Primary or secondary school	60 (13.5)
	Postschool qualification	386 (86.5)
**Main language (n=433), n (%)**		
	English	421 (97.2)
	Other	12 (2.8)
**Socio-Economic Indexes for Areas (n=434), n (%)**		
	Quintiles 1-2	104 (24)
	Quintiles 3-5	330 (76)

Respondents’ mean age was around the age when most women experience menopause [29]. Compared with the general Australian population, the proportions with postschool qualifications (386/446, 86.5% vs 63%) and living in the 3 most advantaged SEIFA quintile areas (330/434, 76% vs 60%) were much higher [30] suggesting that most respondents were well educated and living in relatively advantaged areas. Almost all (421/433, 97.2%) reported that English was their main language.

Responses to the questions about the videos are shown in Table 2.

**Table 2:** Respondents’ views about the videos.

Responses	Video 1^a^ (n=386), n (%)	Video 2^b^ (n=373), n (%)	Video 3^c^ (n=363), n (%)	Video 4^d^ (n=353), n (%)
Information was very easy to understand	355 (92)	341 (91)	334 (92)	321 (91)
All or some information was new	137 (36)	213 (57)	154 (42)	233 (66)
Very or quite likely to use the tips	271 (70)	331 (89)	307 (85)	298 (84)
Would recommend the video to others	235 (61)	263 (70)	244 (67)	232 (66)

^a^What is menopause?

^b^How to stay healthy after menopause?

^c^Slowing weight gain to reduce joint pain.

^d^Maintaining weight for joint health.

Almost all respondents (from 321/353, 91% to 334/363, 92%) agreed that the information in the videos was “very easy to understand.” The proportions of respondents reporting that all or some of the information provided in the video was new to them varied between one-third (137/386, 36%) for the video explaining menopause and two-thirds (233/353, 66%) for the video explaining how maintaining weight benefits joint health. The reported likelihood of using the practical tips offered in the videos was lowest for the video explaining menopause. For the other 3 videos, the vast majority (from 271/386, 70% to 331/373, 89%) stated that they were very or quite likely to use the tips. Around two-thirds (between 235/386, 61% and 263/373, 70%) of respondents stated that they would recommend the videos to others.

Education-level group comparisons revealed few differences in opinions about the videos, except that women with lower education were more likely than those with higher education to state that they would recommend the 2 joint health videos to others (36/45, 80% vs 208/318, 65%; *P*=.051 for video 3; and 36/44, 80% vs 197/309, 64%; *P*=.04 for video 4). There were no differences between women living in the least advantaged areas (SEIFA quintile areas 1 and 2) and those living in the most advantaged areas (SEIFA quintile areas 3, 4 and 5) in their responses to any of the questions about the 4 videos.

## Discussion

### Principal Findings

Menopause is ubiquitous at midlife, and weight gain and joint pain are common in women as they age [31,32]. High-quality, accessible, and evidence-based information is important to allow women to optimize their health in midlife and improve their chances of healthy aging. However, accessing evidence-based and practical information about strategies in midlife to optimize health in older age can be difficult, especially for those with low literacy or from culturally and linguistically diverse backgrounds. To bridge this gap, we developed short, animated videos with practical tips on self-management of menopausal symptoms and joint pain. Evaluation of these videos showed that most women found them easy to understand, learned something new from watching them, planned to use the practical tips they offered, and were likely to recommend them to other women.

Socioeconomic disadvantage is known to be linked to poor health literacy, that is, the capacity to access, understand, appraise, and apply health information [33]. The videos were developed specifically to meet the needs of women who may have limited health literacy and who therefore may not be reached by health-promoting messages on existing websites [19]. However, since most survey respondents were well educated and living in socioeconomically advantaged areas, they were not representative of the target population. This likely reflects the known challenges of selection bias in survey research where people with higher social status and healthier lifestyles are overrepresented [34].

Despite this potential selection bias, the finding that there were no significant differences between the more or less educated groups or between those living in more or less socioeconomically advantaged areas in the proportions reporting that they learned something new from watching the videos suggests that health promotion messages delivered in short video format may benefit a cross-section of women in midlife. This supports the conclusion of a systematic review comparing the impact of video animations with other formats of information delivery on patient knowledge, attitudes, and behaviors, that “video animations show promise as patient information tools, particularly for effects on knowledge” [21].

Health behaviors occur in the context of complex interactions between social, cultural, and economic factors. Most respondents stated that they acquired new knowledge from the videos and intended to use the tips in the videos. While increased knowledge alone might have a limited effect on health behaviors, motivation, and intention are known to be powerful drivers of behavior change [35,36]. The new knowledge respondents reported acquiring from the videos combined with their intention to use the tips in the videos suggest that they may contribute to positive health behavior change among women in midlife.

A limitation of this study was that the proportions of respondents with postschool qualifications and living in the 3 most advantaged SEIFA quintile areas were much higher than in the general population, suggesting selection bias. This may in part be because of the method of recruitment, which included posts on social media and in a women’s health organization’s web-based newsletter that is distributed to women who have opted to receive it. Self-selection and lack of representation of people with limited or no access to the internet are known to contribute to selection bias in web-based surveys, including this survey [37]. A systematic review of strategies to reach hard-to-reach populations in medical research, including socially disadvantaged groups, concluded that they should include researchers and research institutions acknowledging the need for extended timeframes, planning for higher resourcing costs, and recruiting via community partnerships [38]. Another limitation of the study was that some details may have been lost by dichotomizing data.

The videos are available in English, Vietnamese, and Simplified Chinese and currently reside on the website of the Centre for Research Excellence Women and Non-communicable Diseases [39]. Based on the positive findings from this evaluation study, a multipronged dissemination strategy is used to make these and other videos that are currently being developed accessible to as many women as possible. They include publishing a piece in a web-based publication that reaches more than 60,000 medical practitioners in Australia [40]. In this piece, we describe the videos and explain how they can be used as a tool for general practitioners to empower midlife women with knowledge to help them stay healthy for longer, especially those with limited health literacy. We are also making the videos available on government-funded health information websites including Health Translations [41], which has a free library of multilingual health and well-being information. Pending funding, we are also planning to evaluate the videos among Vietnamese- and Chinese-speaking women through interpreter-assisted focus groups.

### Conclusion

This study suggests that short, animated explanatory videos about health self-management strategies in midlife to improve the chance of healthy aging are perceived as accessible, acceptable, easy to understand, and useful by women and associated with high rates of intention to change health behaviors. Future research is needed to determine if and for whom this is followed by more positive health behaviors.

## Abbreviations

SEIFA: Socioeconomic Indexes for Areas

## Data Availability

The datasets generated during and/or analyzed during this study are available from the corresponding author on reasonable request.
